# Supplementary material for: Parenting under the triple burden of violence, depression, and poor diet quality: An intergenerational mother–child syndemic in Nepal
Source: PLOS Glob Public Health. 2026 Feb 18;6(2):e0005977. doi: 10.1371/journal.pgph.0005977 (PMC12915984; doi:10.1371/journal.pgph.0005977)
Supplement: S1 Text — (DOCX) [file pgph.0005977.s001.docx]

Supplementary Appendices Table of Contents

[Fig A. DAG Representing Paths between Maternal Exposures (IPV, Depression, Dietary Iron Intake) and Child Diarrhea Morbidities 2](#_Toc221274526)

[Table A. Covariate Operationalization Strategy using the 2002 Nepal Demographic and Health Survey (DHS) 3](#_Toc221274527)

[Equation A. Regression Model for the Fully Saturated Multivariable Logistic Regression Model 6](#_Toc221274528)

[Equation B. Regression Model for the Fully Saturated Multivariable Linear Probability Model 6](#_Toc221274529)

[Text: Relative Excess Risk Due to Interaction (RERI) for Three-Term Interactions 7](#_Toc221274530)

[Table B. Results and Interpretation of the RERI 8](#_Toc221274531)

[Table C. Summary of Sensitivity Models and Related Results 9](#_Toc221274532)

[Table D. Sensitivity Models Using Any IPV, Dietary Consumption of Heme Sources, and PHQ-9 Depression Symptoms 10](#_Toc221274533)

[Table E. Sensitivity Models Using Physical IPV, Dietary Consumption of Heme Sources, and PHQ-9 Depression Symptoms 12](#_Toc221274534)

[Table F. Sensitivity Models Using Emotional IPV, Dietary Consumption of Heme Sources, and PHQ-9 Depression Symptoms 14](#_Toc221274535)

[Table G. Sensitivity Models Using Sexual IPV, Dietary Consumption of Heme Sources, and PHQ-9 Depression Symptoms 16](#_Toc221274536)

# Fig A. DAG Representing Paths between Maternal Exposures (IPV, Depression, Dietary Iron Intake) and Child Diarrhea Morbidities


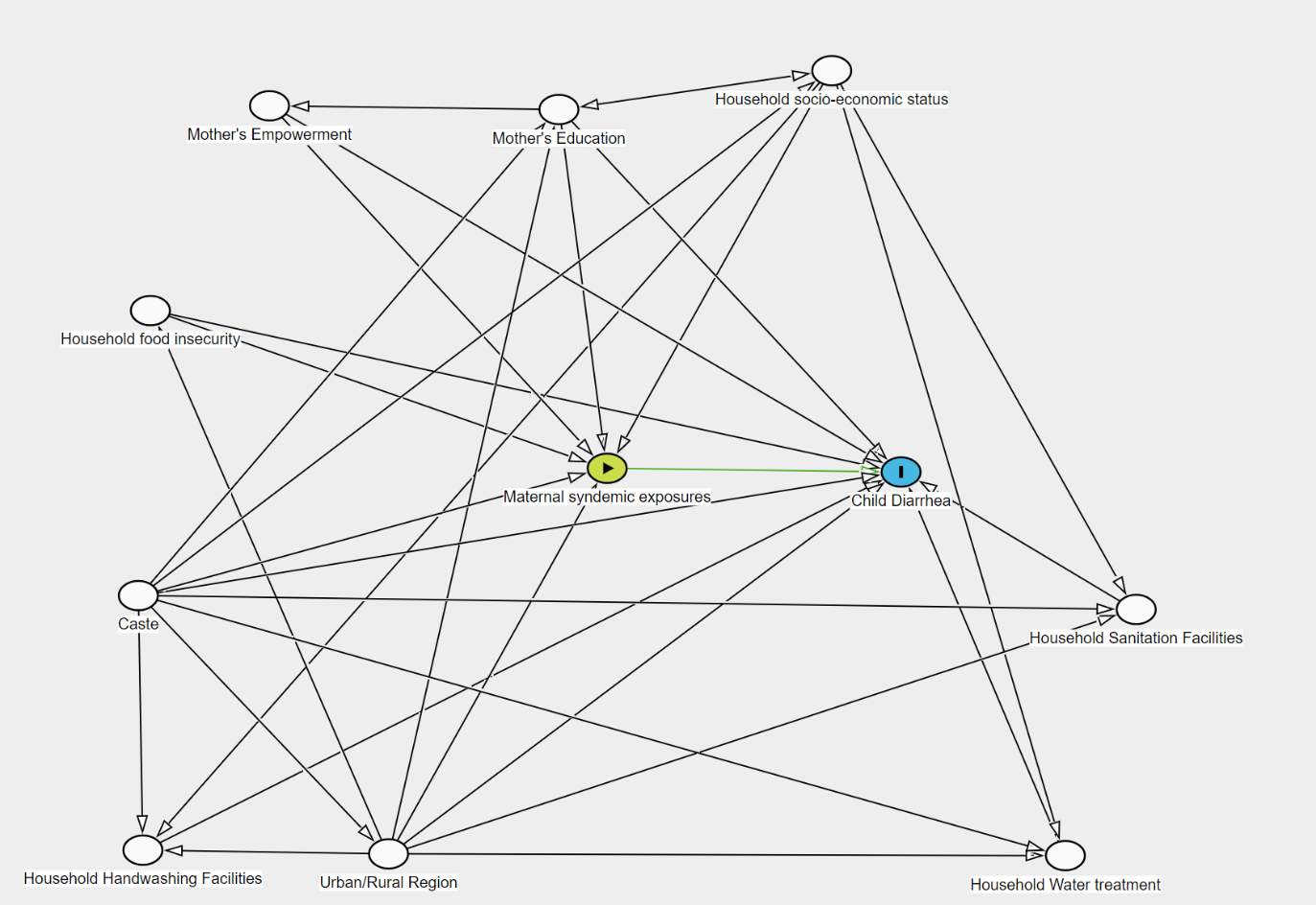


**Legend:**


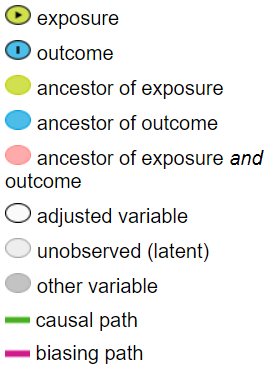

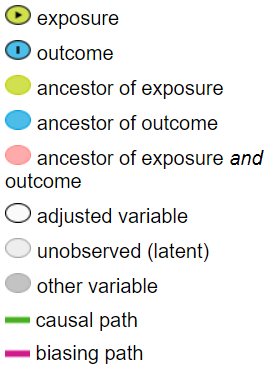


To estimate the total effect of maternal syndemic exposures on child diarrhea, the minimal sufficient adjustment set of covariates included: caste, household handwashing facilities, household sanitation facilities, household water treatment, household food insecurity, household socioeconomic status, mother's education, mother's empowerment, urban/rural region. However, we conducted a subpopulation analysis to assess the role of the context. Based on this DAG, controlling for household wealth (a proxy for socioecojomic status) does not bias the effect being estimated. The green path depicts the total causal effect of each maternal exposure on child diarrhea. The white circles depict variables adjusted for in the statistical analyses. All estimated parameters in the present research represent total adjusted effects.

| **Covariate** | **DHS Measurement Approach** | **Operationalization** |
| --- | --- | --- |
| Food Insecurity | Food Insecurity Experience Scale (FIES):  Now I would like to ask you some questions about food. During the last 12 months, was there a time when:   - You or others in your household worried about not having enough food to eat because of a lack of money or other resources? - Still thinking about the last 12 months, was there a time when you or others in your household were unable to eat healthy and nutritious food because of a lack of money or other resources? - Was there a time when you or others in your household ate only a few kinds of foods because of a lack of money or other resources? - Was there a time when you or others in your household had to skip a meal because there was not enough money or other resources to get food? - Still thinking about the last 12 months, was there a time when you or others in your household ate less than you thought you should because of a lack of money or other resources? - Was there a time when your household ran out of food because of a lack of money or other resources? - Was there a time when you or others in your household were hungry but did not eat because there was not enough money or other resources for food? - Was there a time when you or others in your household went without eating for a whole day because of a lack of money or other resources? | Score count |

# Table A. Covariate Operationalization Strategy using the 2002 Nepal Demographic and Health Survey (DHS)

| Women’s empowerment | Who usually makes decisions about health care for yourself: you, your (husband/partner), you and your (husband/partner) jointly, or someone else?  Who usually makes decisions about making major household purchases?  Who usually makes decisions about visits to your family or relatives? | Binary  Women who rely solely on their spouse or another family member to make decisions related to their own health care, large household purchases, and visits to family or relatives were coded as 1. Joint or sole decision making in all three domains was coded as 0 (ref). |
| --- | --- | --- |
| Women’s education | What is the highest level your attended? : primary, secondary, or higher?” | Binary  All women with incomplete primary school education were coded as 1, otherwise women with primary school completed or higher were coded as 0. |
| Residence type | The DHS indicates whether each respondent’s residence is in an urban or rural location | Binary  Rural was coded as 1 and urban was coded as 0. |
| Caste | What is your ethnicity?   - Hill brahmin - Hill chhetri - Terai brahmin/chettri - Other terai caste - Hill dalit - Terai dalit - Newar - Hill janajati - Terai janajati - Muslim | Binary  The upper castes (Brahmin and Chettri) were coded as 1 and the lower cases were coded as 0. |
| Household water treatment | What do you usually do to make the water safer to drink?   - Boil - Add bleach/chlorine - Strain through a cloth - Use water filter (ceramic/Sand/composite/etc.) - Solar disinfection - Let it stand and settle | Binary  Use of an appropriate water treatment method (boiling, solar disinfection, or adding bleach, chlorine, ceramic, sand or other filters) was coded as 1, otherwise other responses were coded as 0. |
| Household sanitation facilities | What kind of toilet facility do members of your household usually use?   - Flush to piped sewer system - Flush to septic tank - Flush to pit latrine - Flush to somewhere else - Flush, don't know where - Ventilated improved pit latrine - Pit latrine with slab - Pit latrine without slab/open pit - Composting toilet (slab) - Composting toilet (without slab) - Bucket toilet - Biogas attached toilet - No facility/bush/field | Ordinal  Basic sanitation facilities (flush to piped sewer system/septic tank/pit latrine/other, ventilated improved pit, pit latrine with slab, composting toilet) were coded as 0, limited sanitation facilities (shared toilet facilities) were coded as 1 and unimproved sanitation facilities (flush to somewhere else, pit latrine without a slab/open pit, bucket toilet, hanging toilet/latrine) or open defecation were coded as 2 |
| Household handwashing facilities | We would like to learn about the places that households use to wash their hands. Can you please show me where members of your household most often wash their hands? | Binary  Basic handwashing facilities (having a fixed or mobile place for handwashing in dwelling with soap and water) were coded as 1 and limited (not having a fixed or mobile place for handwashing in dwelling or without soap and water) or no handwashing facilities were coded as 0. |

# Equation A. Regression Model for the Fully Saturated Multivariable Logistic Regression Model

$$\ln\left( \frac{p_{i}}{1-p_{i}} \right)=\beta_{0}+\beta_{1}x_{1}+\beta_{2}x_{2}+\beta_{3}x_{3}+\beta_{4}x_{1}x_{2}+\beta_{5}x_{1}x_{3}*\beta_{6}x_{3}x_{2}+\beta_{7}x_{1}x_{2}x_{3}+X\beta$$

For the multiplicative scale (logistic regression) in Equation 1:

$$\beta_{7}=\frac{{OR}_{111}}{{OR}_{110}{OR}_{101}{OR}_{011}{OR}_{001}{OR}_{010}{OR}_{100}}$$

The superscripts represent each syndemic exposure, with 1 denoting the presence of binary exposures or a one-unit increase of the numeric exposure.

# Equation B. Regression Model for the Fully Saturated Multivariable Linear Probability Model

$$Y=\beta_{0}+\beta_{1}x_{1}+\beta_{2}x_{2}+\beta_{3}x_{3}+\beta_{4}x_{1}x_{2}+\beta_{5}x_{1}x_{3}*\beta_{6}x_{3}x_{2}+\beta_{7}x_{1}x_{2}x_{3}+X\beta$$

For the additive scale (linear probability model) in Equation 2:

$\beta_{7}=$ RR_111_-RR_110_-RR_101_-RR_011_+RR_100_+RR_010_+RR_001_-RR_000_

The superscripts represent each syndemic exposure, with 1 denoting the presence of binary exposures or a one unit increase of the numeric exposure.

Legend:

Y is the child diarrhea outcome

$p_{i}$ probability that a child has diarrhea morbidities.

$\beta_{1}$coefficient for maternal IPV exposure.

$\beta_{2}$ coefficient for maternal depression score.

$\beta_{3}$ coefficient for maternal dietary intake of iron-rich foods.

$\beta_{4}$ joint effect for double exposure: IPV & depression score.

$\beta_{5}$ joint effect for double exposure: IPV & dietary intake of iron-rich foods.

$\beta_{6}$ joint effect for double exposure: depression score & dietary intake of iron-rich foods.

$\beta_{7}$ joint effect for triple exposure: IPV, depression score, & dietary intake of iron-rich foods.

Xβ is the matrix set of confounding variables and their coefficients.

OR is the odds ratio, computed by exponentiating the estimates from Equation 1.

RR is the relative risk, which is the beta estimate from Equation 2.

*Analytical Strategy for Assessing the Environment*

A subpopulation analysis involving the two household-wealth groups (most disadvantaged and more advantaged) was conducted for the multiplicative and additive scale regression models. The models expressed in Equation 1 and Equation 2 were stratified by the binary household wealth contextual variable using the *svy, subpop(): logit* and *svy, subpop(): regresss* commands. The significance of the multiplicative (from Equation 1) and additive interactions (from Equation 2) were assessed in the most disadvantaged households (poorest and poor household wealth) and in more advantaged households (middle, less poor, least poor house wealth).

# Text: Relative Excess Risk Due to Interaction (RERI) for Three-Term Interactions

Based on existing guidance ^[60,70]^, additive interaction for a three-term interaction, calculated using the logistic regression scale, is given by

RERI_3_ =OR_111_-OR_110_-OR_101_-OR_011_+OR_100_+OR_010_+OR_001_-OR_000_ [Equation 1]

Equation 1 assumes the odds ratio approximates the relative risk, meaning the outcome is relatively rare (typically less than 5% prevalence) ^[60,78]^. Only the direction of the RERI is interpreted, not the magnitude of the underlying interaction: Additive interaction: RERI >0, Negative interaction: RERI <0, No iteration: RERI = 0. Vanderweele (2015) provides further guidance for assessing additive interactions involving continuous exposures, wherein one can calculate the RER comparing two different levels of exposure for a continuous term in the interaction ^[60]^. In this case, the mental health depression score (measured using the PHQ-10) is numerical. Thus, the RERI was calculated under two conditions (1) one single unit increase in depression score and (2) a 10 unit increase in depression from 0 to 10. A depression score of 0 to 10 is meaningful as this corresponds to a mother with ‘no depression’ to ‘moderate/severe depression.

Using the aOR from Model 1, Table 2, the RERI for a one unit increase in all interactions was computed in Stata as follows ^[70]^:

nlcom exp(_b[1.dv_any_12m#0.nt_iron_dk#c.mth22] + _b[0.nt_iron_dk#c.mth22] + _b[1.dv_any_12m#0.nt_iron_dk] + _b[1.dv_any_12m#c.mth22] + _b[0.nt_iron_dk] + _b[mth22] + _b[1.dv_any_12m] )-exp(_b[0.nt_iron_dk#c.mth22] + _b[mth22] + _b[0.nt_iron_dk])-exp( _b[1.dv_any_12m#0.nt_iron_dk] + _b[1.dv_any_12m] + _b[0.nt_iron_dk] )-exp(_b[1.dv_any_12m#c.mth22] + _b[1.dv_any_12m] + _b[mth22]) + exp( _b[1.dv_any_12m]) + exp(_b[0.nt_iron_dk]) +exp(_b[mth22]) – 1 [Equation 2]

Where:

dv_any_12m = estimated b for intimate partner violence exposure vs no exposure

0.nt_iron = estimated b for not consuming dietary iron vs consuming dietary iron

c.mth22 = depression score using the PHQ-9 (one unit increase)

Using the aOR from Model 1, Table 2, the RERI for 10 unit increase in depression score (from 0 10 10) was computed in Stata as:

nlcom exp((mh1-mh0)*_b[1.dv_any_12m#0.nt_iron_dk#c.mth22] + (mh1-mh0)*_b[0.nt_iron_dk#c.mth22] + _b[1.dv_any_12m#0.nt_iron_dk] + (mh1-mh0)* _b[1.dv_any_12m#c.mth22] + _b[0.nt_iron_dk] + (mh1-mh0)*_b[mth22] + _b[1.dv_any_12m] )-exp( (mh1-mh0)*_b[0.nt_iron_dk#c.mth22] + (mh1-mh0)* _b[mth22] + _b[0.nt_iron_dk])-exp( _b[1.dv_any_12m#0.nt_iron_dk] + _b[1.dv_any_12m] + _b[0.nt_iron_dk] )-exp((mh1-mh0)*_b[1.dv_any_12m#c.mth22] + _b[1.dv_any_12m] + (mh1-mh0)*_b[mth22]) + exp( _b[1.dv_any_12m]) + exp(_b[0.nt_iron_dk]) +exp((mh1-mh0)*_b[mth22]) – 1 [Equation 3]

where mh1=10 and mh0=0 (representing a 10-unit increase)

# Table B. Results and Interpretation of the RERI

| **RERI Type** | **Results** | **Interpretation** |
| --- | --- | --- |
| RERI for a 1-point increase in depression | RERI_3_=*.*1702413  95% CI: (-0.09, 0.43)  *P* value=0.202 | RERI suggests an additive interaction given the value is >0 and most of the confidence interval spans the positive scale. However, the *P* value is nonsignificant. Given the outcome of child diarrhea is not rare, the RERI is interpreted with caution. |
| RERI for a 10-point increase in depression | RERI_3_= 3.09  95% CI: (-0.34, 6.52)  *P* value=0.08 | Greater evidence for a positive interaction given the value is >0 and most of the confidence interval spans the positive scale. However, the *P* value is marginally nonsignificant. Given the outcome of child diarrhea is not rare, the RERI is interpreted with caution. |

# Table C. Summary of Sensitivity Models and Related Results

| Syndemic Exposures | | | Interpretation for Multiplicative Model | Interpretation for Additive Model |
| --- | --- | --- | --- | --- |
| IPV | Dietary iron | Depression |  |  |
| Any | Heme sources | PHQ-9 | Omnibus test for the model was significant.  For more disadvantaged households: Joint effects for the three-term interaction became more significant (*P=*0.039).  For less disadvantaged households: Joint effects for the three-term interaction was not significant. | For more disadvantaged households: Joint effects three-term interaction was marginally insignificant (*P=*0.096).  For less disadvantaged households: Joint effects three-term interaction was not significant. |
| Physical | Heme sources | PHQ-9 | Omnibus test for the model was significant.  For more disadvantaged households: Joint effects for the three-term interaction became more significant (*P=*0.014) and point estimate more extreme (aOR: 1.352).  For less disadvantaged households: Joint effects three-term interaction was not significant. | For less disadvantaged households: Joint effects three-term interaction became more significant (*P=*0.039).  For less disadvantaged households: Joint effects three-term interaction was not significant. |
| Emotional | Heme sources | PHQ-9 | Omnibus test for the model was significant.  For more disadvantaged households: Joint effects for the three-term interaction became more significant (*P=*0.042) and point estimate more extreme (aOR: 1.329).  For less disadvantaged households: Joint effects for the three-term interaction was not significant. | For more disadvantaged households: Joint effects three-term interaction was not significant.  For less disadvantaged households: Joint effects three-term interaction was not significant. |
| Sexual | Heme sources | PHQ-9 | Omnibus test for the model was significant.  Joint effects three-term interaction was not significant for more disadvantaged and less disadvantaged households. | Joint effects three-term interaction was not significant for more disadvantaged and less disadvantaged households. |

# Table D. Sensitivity Models Using Any IPV, Dietary Consumption of Heme Sources, and PHQ-9 Depression Symptoms

|  | Model 1a: Multiplicative Scale Most Disadvantaged Households |  | Model 1b: Additive Scale Most Disadvantaged Households |  | Model 2a: Multiplicative Scale Less Disadvantaged Households |  | Model 2b: Additive Scale Less Disadvantaged Households |
| --- | --- | --- | --- | --- | --- | --- | --- |
|  | aOR *P* value 95% CI |  | B *P* value 95% CI |  | aOR *P* value 95% CI |  | B *P* value 95% CI |
| Mother experienced any IPV in the past 12 months |  |  |  |  |  |  |  |
| (ref: unexposed to IPV) | 0.779 |  | -0.016 |  | 4.973 |  | 0.199 |
|  | 0.696 |  | 0.759 |  | 0.063 |  | 0.168 |
|  | [0.223, 2.724] |  | [-0.116, 0.085] |  | [0.919, 26.922] |  | [-0.084, 0.481] |
| Mother’s depression score (one unit increase) | 1.107 |  | 0.011 |  | 1.089 |  | 0.008 |
|  | 0.063 |  | 0.156 |  | 0.285 |  | 0.335 |
|  | [0.995, 1.233] |  | [-0.004, 0.026] |  | [0.931, 1.274] |  | [-0.009, 0.026] |
| Mother did not consume heme iron sources |  |  |  |  |  |  |  |
| (ref: consumed heme iron source(s)) | 0.788 |  | -0.016 |  | 0.677 |  | -0.025 |
|  | 0.486 |  | 0.536 |  | 0.348 |  | 0.372 |
|  | [0.402, 1.543] |  | [-0.067, 0.035] |  | [0.300, 1.530] |  | [-0.079, 0.030] |
| Mother experienced any IPV in the past 12 months + Depression score |  |  |  |  |  |  |  |
|  | 0.897 |  | -0.011 |  | 0.779 |  | -0.027 |
|  | 0.180 |  | 0.196 |  | 0.144 |  | 0.156 |
|  | [0.764, 1.052] |  | [-0.029, 0.006] |  | [0.557, 1.089] |  | [-0.063, 0.010] |
| Mother did not consume heme iron sources + Depression score |  |  |  |  |  |  |  |
|  | 0.926 |  | -0.008 |  | 1.045 |  | 0.002 |
|  | 0.279 |  | 0.336 |  | 0.646 |  | 0.827 |
|  | [0.806, 1.064] |  | [-0.025, 0.009] |  | [0.866, 1.260] |  | [-0.018, 0.022] |
| Mother did not consume heme iron sources + Mother Experienced any IPV in past 12 months |  |  |  |  |  |  |  |
|  | 0.401 |  | -0.048 |  | 0.669 |  | -0.079 |
|  | 0.289 |  | 0.395 |  | 0.705 |  | 0.644 |
|  | [0.074, 2.176] |  | [-0.160, 0.063] |  | [0.083, 5.391] |  | [-0.415, 0.256] |
| Mother experienced any IPV in the past 12 months + Mother did not consume heme iron sources + Depression score |  |  |  |  |  |  |  |
|  | 1.255 | * | 0.018 |  | 1.162 |  | 0.018 |
|  | 0.039 |  | 0.096 |  | 0.430 |  | 0.411 |
|  | [1.012, 1.558] |  | [-0.003, 0.040] |  | [0.800, 1.689] |  | [-0.026, 0.063] |
|  |  |  |  |  |  |  |  |
| Omnibus model fit test | F(16, 447)=2.03 |  | F(16, 447)=1.53 |  | F(16, 477)=1.79 |  | F(16, 447)=1.38 |
| Number of observations | 1139 |  | 1139 |  | 807 |  | 807 |

* *P*<.05. All models controlled for the same set of covariates deemed to be confounders based on the DAG presented in Figure 5: Food insecurity urban/rural residence, women’s empowerment, women’s education, caste membership, household water treatment, and water and sanitation facility access (handwashing and sanitation).

# Table E. Sensitivity Models Using Physical IPV, Dietary Consumption of Heme Sources, and PHQ-9 Depression Symptoms

|  | Model 1a: Multiplicative Scale Most Disadvantaged Households |  | Model 1b: Additive Scale Most Disadvantaged Households |  | Model 2a: Multiplicative Scale Less Disadvantaged Households |  | Model 2b: Additive Scale Less Disadvantaged Households |
| --- | --- | --- | --- | --- | --- | --- | --- |
|  | aOR *P* value 95% CI |  | B *P* value 95% CI |  | aOR *P* value 95% CI |  | B *P* value 95% CI |
| Mother experienced physical IPV in the past 12 months | 0.777 |  | -0.015 |  | 0.816 |  | -0.018 |
| (ref: unexposed to physical IPV) | 0.727 |  | 0.798 |  | 0.829 |  | 0.828 |
|  | [0.188, 3.211] |  | [-0.134, 0.103] |  | [0.128, 5.187] |  | [-0.184, 0.148] |
|  | 1.107 |  | 0.010 |  | 1.038 |  | 0.004 |
| Mother’s depression score (one unit increase) | 0.053 |  | 0.133 |  | 0.640 |  | 0.612 |
|  | [0.999, 1.227] |  | [-0.003, 0.024] |  | [0.888, 1.214] |  | [-0.012, 0.021] |
|  |  |  |  |  |  |  |  |
| Mother did not consume heme iron sources | 0.797 |  | -0.014 |  | 0.518 |  | -0.050 |
| (ref: consumed heme iron source(s)) | 0.489 |  | 0.564 |  | 0.128 |  | 0.144 |
|  | [0.418, 1.519] |  | [-0.063, 0.034] |  | [0.222, 1.209] |  | [-0.117, 0.017] |
|  |  |  |  |  |  |  |  |
| Mother experienced physical IPV in the past 12 months + Depression score | 0.884 |  | -0.012 |  | 0.985 |  | -0.003 |
|  | 0.153 |  | 0.159 |  | 0.919 |  | 0.845 |
|  | [0.747, 1.047] |  | [-0.029, 0.005] |  | [0.731, 1.327] |  | [-0.028, 0.023] |
|  |  |  |  |  |  |  |  |
| Mother did not consume heme iron sources + Depression score | 0.923 |  | -0.008 |  | 1.141 |  | 0.012 |
|  | 0.217 |  | 0.258 |  | 0.152 |  | 0.222 |
|  | [0.813, 1.048] |  | [-0.023, 0.006] |  | [0.953, 1.366] |  | [-0.007, 0.032] |
|  |  |  |  |  |  |  |  |
| Mother did not consume heme iron sources + Mother Experienced physical IPV in past 12 months | 0.264 |  | -0.070 |  | 4.000 |  | 0.140 |
|  | 0.228 |  | 0.299 |  | 0.251 |  | 0.290 |
|  | [0.030, 2.307] |  | [-0.202, 0.062] |  | [0.373, 42.904] |  | [-0.120, 0.400] |
|  |  |  |  |  |  |  |  |
| Mother experienced physical IPV in the past 12 months + Mother did not consume heme iron sources + Depression score | 1.352 | * | 0.025 | * | 0.819 |  | -0.020 |
|  | 0.014 |  | 0.039 |  | 0.311 |  | 0.293 |
|  | [1.063, 1.721] |  | [0.001, 0.049] |  | [0.557, 1.205] |  | [-0.057, 0.017] |
|  |  |  |  |  |  |  |  |
| Omnibus model fit test | F(16, 447)=2.14 |  | F(16, 477)=1.64 |  | F(16, 477)=1.81 |  | F(16, 447)=1.47 |
| Number of observations | 1139 |  | 1139 |  | 807 |  | 807 |

* *P*<.05. All models controlled for the same set of covariates deemed to be confounders based on the DAG presented in Figure 5: Food insecurity urban/rural residence, women’s empowerment, women’s education, caste membership, household water treatment, and water and sanitation facility access (handwashing and sanitation).

# Table F. Sensitivity Models Using Emotional IPV, Dietary Consumption of Heme Sources, and PHQ-9 Depression Symptoms

|  | Model 1a: Multiplicative Scale Most Disadvantaged Households |  | Model 1b: Additive Scale Most Disadvantaged Households |  | Model 2a: Multiplicative Scale Less Disadvantaged Households |  | Model 2b: Additive Scale Less Disadvantaged Households |
| --- | --- | --- | --- | --- | --- | --- | --- |
|  | aOR *P* value 95% CI |  | B *P* value 95% CI |  | aOR *P* value 95% CI |  | B *P* value 95% CI |
| Mother experienced emotional IPV in the past 12 months | 1.439 |  | 0.027 |  | 8.349 | * | 0.322 |
| (ref: unexposed to emotional IPV) | 0.666 |  | 0.703 |  | 0.019 |  | 0.082 |
|  | [0.275, 7.527] |  | [-0.114, 0.169] |  | [1.430, 48.744] |  | [-0.041, 0.685] |
|  | 1.073 |  | 0.008 |  | 1.048 |  | 0.005 |
| Mother’s depression score (one unit increase) | 0.115 |  | 0.192 |  | 0.552 |  | 0.553 |
|  | [0.983, 1.172] |  | [-0.004, 0.019] |  | [0.897, 1.226] |  | [-0.010, 0.019] |
|  |  |  |  |  |  |  |  |
| Mother did not consume heme iron sources | 0.761 |  | -0.020 |  | 0.779 |  | -0.017 |
| (ref: consumed heme iron source(s)) | 0.406 |  | 0.428 |  | 0.535 |  | 0.557 |
|  | [0.400, 1.449] |  | [-0.068, 0.029] |  | [0.353, 1.717] |  | [-0.073, 0.039] |
|  |  |  |  |  |  |  |  |
| Mother experienced emotional IPV in the past 12 months + Depression score | 0.873 |  | -0.012 |  | 0.810 |  | -0.030 |
|  | 0.156 |  | 0.159 |  | 0.196 |  | 0.149 |
|  | [0.723, 1.053] |  | [-0.028, 0.005] |  | [0.588, 1.116] |  | [-0.070, 0.011] |
|  |  |  |  |  |  |  |  |
| Mother did not consume heme iron sources + Depression score | 0.981 |  | -0.002 |  | 1.044 |  | 0.003 |
|  | 0.742 |  | 0.714 |  | 0.639 |  | 0.732 |
|  | [0.873, 1.102] |  | [-0.016, 0.011] |  | [0.872, 1.249] |  | [-0.014, 0.021] |
|  |  |  |  |  |  |  |  |
| Mother did not consume heme iron sources + Mother Experienced emotional IPV in past 12 months | 0.079 | * | -0.106 |  | 0.393 |  | -0.198 |
|  | 0.043 |  | 0.167 |  | 0.427 |  | 0.376 |
|  | [0.007, 0.927] |  | [-0.257, 0.044] |  | [0.039, 3.959] |  | [-0.637, 0.241] |
|  |  |  |  |  |  |  |  |
| Mother experienced emotional IPV in the past 12 months + Mother did not consume heme iron sources + Depression score | 1.329 | * | 0.015 |  | 1.184 |  | 0.028 |
|  | 0.042 |  | 0.166 |  | 0.352 |  | 0.262 |
|  | [1.010, 1.747] |  | [-0.006, 0.037] |  | [0.830, 1.689] |  | [-0.021, 0.077] |
|  |  |  |  |  |  |  |  |
| Omnibus model fit test | F(16, 447)=2.02 |  | F(16, 447)=1.76 |  | F(16, 447)=1.98 |  | F(16, 447)=1.43 |
| Number of observations | 1139 |  | 1139 |  | 807 |  | 807 |

**P* <.05. All models controlled for the same set of covariates deemed to be confounders based on the DAG presented in Figure 5: Food insecurity urban/rural residence, women’s empowerment, women’s education, caste membership, household water treatment, and water and sanitation facility access (handwashing and sanitation).

# Table G. Sensitivity Models Using Sexual IPV, Dietary Consumption of Heme Sources, and PHQ-9 Depression Symptoms

|  | Model 1a: Multiplicative Scale Most Disadvantaged Households |  | Model 1b: Additive Scale Most Disadvantaged Households |  | Model 2a: Multiplicative Scale Less Disadvantaged Households |  | Model 2b: Additive Scale Less Disadvantaged Households |
| --- | --- | --- | --- | --- | --- | --- | --- |
|  | aOR *P* value 95% CI |  | B *P* value 95% CI |  | aOR *P* value 95% CI |  | B *P* value 95% CI |
| Mother experienced sexual IPV in the past 12 months | 1.750 |  | 0.097 |  | 43.578 |  | 0.436 |
| (ref: unexposed to sexual IPV) | 0.522 |  | 0.407 |  | 0.050 |  | 0.090 |
|  | [0.314, 9.736] |  | [-0.132, 0.326] |  | [1.006, 1887.496] |  | [-0.068, 0.939] |
|  | 1.044 |  | 0.004 |  | 1.046 |  | 0.005 |
| Mother’s depression score (one unit increase) | 0.299 |  | 0.381 |  | 0.546 |  | 0.535 |
|  | [0.962, 1.132] |  | [-0.005, 0.013] |  | [0.905, 1.209] |  | [-0.010, 0.019] |
|  |  |  |  |  |  |  |  |
| Mother did not consume heme iron sources | 0.709 |  | -0.023 |  | 0.667 |  | -0.033 |
| (ref: consumed heme iron source(s)) | 0.294 |  | 0.332 |  | 0.341 |  | 0.339 |
|  | [0.373, 1.349] |  | [-0.070, 0.024] |  | [0.289, 1.538] |  | [-0.099, 0.034] |
|  |  |  |  |  |  |  |  |
| Mother experienced sexual IPV in the past 12 months + Depression score | 0.965 |  | -0.006 |  | 0.598 |  | -0.043 |
|  | 0.771 |  | 0.731 |  | 0.075 |  | 0.065 |
|  | [0.759, 1.227] |  | [-0.038, 0.027] |  | [0.340, 1.053] |  | [-0.088, 0.003] |
|  |  |  |  |  |  |  |  |
| Mother did not consume heme iron sources + Depression score | 0.983 |  | -0.002 |  | 1.083 |  | 0.008 |
|  | 0.741 |  | 0.701 |  | 0.336 |  | 0.349 |
|  | [0.885, 1.091] |  | [-0.012, 0.008] |  | [0.921, 1.273] |  | [-0.009, 0.025] |
|  |  |  |  |  |  |  |  |
| Mother did not consume heme iron sources + Mother Experienced sexual IPV in past 12 months | 0.146 |  | -0.220 |  | 0.548 |  | 0.008 |
|  | 0.160 |  | 0.093 |  | 0.796 |  | 0.984 |
|  | [0.010, 2.149] |  | [-0.476, 0.037] |  | [0.006, 52.812] |  | [-0.798, 0.814] |
|  |  |  |  |  |  |  |  |
| Mother experienced sexual IPV in the past 12 months + Mother did not consume heme iron sources + Depression score | 1.277 |  | 0.031 |  | 0.930 |  | -0.013 |
|  | 0.125 |  | 0.138 |  | 0.830 |  | 0.727 |
|  | [0.934, 1.746] |  | [-0.010, 0.072] |  | [0.482, 1.797] |  | [-0.085, 0.059] |
|  |  |  |  |  |  |  |  |
| Omnibus model fit test | F(16, 447)=2.31 |  | F(16, 447)=1.57 |  | F(16, 447)=2.29 |  | F(16, 447)=1.68 |
| Number of observations | 1139 |  | 1139 |  | 807 |  | 807 |

* *P*<.05All models controlled for the same set of covariates deemed to be confounders based on the DAG presented in Figure 5: Food insecurity urban/rural residence, women’s empowerment, women’s education, caste membership, household water treatment, and water and sanitation facility access (handwashing and sanitation).
