# Supplementary material for: Parenting under the triple burden of violence, depression, and poor diet quality: An intergenerational mother–child syndemic in Nepal
Source: PLOS Glob Public Health. 2026 Feb 18;6(2):e0005977. doi: 10.1371/journal.pgph.0005977 (PMC12915984; doi:10.1371/journal.pgph.0005977)
Supplement: Attachment — Submitted filename: PLOSglobalhealth_review_rl+tk.docx [file pgph.0005977.s002.docx]

This study focuses on identifying the relationship between concurrent exposure of intimate partner violence, depression, and inadequate iron intake among mothers on diarrheal disease among children. The study used a national representative sample of women of reproductive age in Nepal. The findings suggest risk of diarrhea is higher in households with concurrent exposure, and that this is further exacerbated by living in a disadvantaged household. The manuscript is well written and very comprehensive with all major topics addressed effectively. The methods present clear description of source of data, procedures used, and approach, providing solid understanding. However, to build a case for examining this association specifically, the relationship of each variable among mothers with the diarrheal disease in children could be more thoroughly explained. What factors specifically influence the connection between depressive symptoms and diarrhea for example? This is not clear enough in Figure 1. From the text, it seems like the authors are suggesting it’s parenting practices that connect these two, but I think more evidence would be needed to justify this over some confounding factor like water quality or poverty. Furthermore, what theoretical mechanisms suggest there is a syndemic effect of these three, related, issues? The use of the theoretical framework is a strength, but the lack of clarity, particularly in the introduction and figure, to justify a hypothesized relationship is a major limitation that limits our enthusiasm considerably.

**Major comments**

Methodology- The binary categorization of iron intake does not capture adequacy. Consider rewording to avoid misinterpretation since adequacy may have different implications than presence/absence of intake alone. In one table, you use no dietary iron intake. I like this better than claims around adequacy, which would be difficult to interpret based on the measure you used.

Line 218 - Maternal syndemic exposures. Can you provide rationale for the temporality of the explored relationship? This is especially important since some of the results imply directionality, yet iron intake is measured on a shorter time horizon than diarrhea symptoms.

Results- Consider streamlining of results section. Since the central theme of the article is triple exposure, creating groups (IPV+Dep+Fe or something like this) and labelling can improve readability. The use of triply/doubly/singly exposed was hard to follow.

Discussion- Consider linking current findings to the existent literature to further strengthen discussion. Right now, there is a somewhat limited use of citations and not sufficient connection back to the actual findings of the study. Much of the section about potential intervention pathways feels premature based on your findings. This may be more noticeable because the mechanism connecting the exposures with the outcome doesn’t feel clear.

**Minor comments:**

Line 97-98: *These findings indicate a mechanism…via mediators such as diarrheal*. Please add citation/s to support this point.

Line 105-108: *IPV can lead to…leave children more vulnerable to diarrheal disease.* Please add citation/s to support this point.

Line 122-123: *Inadequate dietary intake…healthy parenting practices.* Please add citation/s to support this point.

Line 161-163: *In many LMIC’s…high income counterparts.* Please add citation/s to support this point.

Line 281: A comma seems to be missing between food insecurity and urban/rural residence

Line 397: It is written as “470 of 10,000” instead of 100,000. This appears to be a typographical error and should be corrected for consistency.

Page 23: *Counterfactually, for mothers… (****P=0.00****)…nonsignificant (29.8%, P=0.195).* Could you please correct the p-value and simplify the language for better understanding of this result. We found this to be confusing

*Page 29:* GBV is not spelled out.

Limitations – You mention caste membership and sanitation as covariates in the methods and then discuss it again in the limitations. Please add an explanation of why controlling for these in the analysis was not sufficient.
