# Supplementary material for: Parenting under the triple burden of violence, depression, and poor diet quality: An intergenerational mother–child syndemic in Nepal
Source: PLOS Glob Public Health. 2026 Feb 18;6(2):e0005977. doi: 10.1371/journal.pgph.0005977 (PMC12915984; doi:10.1371/journal.pgph.0005977)
Supplement: Attachment — Submitted filename: Review for Nepal Syndemic Study_NS9252025.docx [file pgph.0005977.s003.docx]

**Review For:** Parenting Under the Triple Burden of Violence, Depression, and Malnutrition: An Intergenerational Mother–Child Syndemic in Nepal

This manuscript addresses an important and novel question by applying syndemic theory to maternal and child health in Nepal. The study uses nationally representative DHS data and rigorous interaction modeling to explore how IPV, maternal depression, and inadequate dietary iron intake cluster and interact to affect child diarrhea outcomes. The theoretical contribution is strong, and the policy relevance is clear. However, the manuscript could do with significant tightening (moving material to an appendix), stronger contextual justification, and clearer alignment between theory, exposures, and outcomes.

**Abstract**

- Novel application of syndemic theory; well-structured research questions; strong methodological rigor.
- Diarrhea is introduced abruptly and not adequately justified as the outcome; conceptual drift between “child health disparities” broadly and “diarrhea” specifically;
- dramatic probability increases need context (CIs, effect sizes). A bit confusing – would benefit from more clarity.

**Introduction & Literature Review**

- Broad, well-referenced coverage of IPV, depression, nutrition, and gender inequities.
- The first two paragraphs (lines 68–87) pack in too much — global child mortality, diarrheal disease burden, services, parenting roles, and gender inequity — without clearly signaling the specific *research problem*. It feels like you are setting up multiple possible papers: one on diarrhea epidemiology, another on maternal mental health, another on gendered nutrition inequities. It could help to condense the background to 2–3 crisp points: (1) diarrhea is a major child mortality driver in LMICs, (2) maternal health and caregiving context strongly influence child vulnerability, (3) three maternal adversities (IPV, depression, inadequate nutrition) may jointly increase risk.
- need more justification for diarrhea vs. other outcomes;
- maternal exposures reviewed in parallel instead of as an interconnected system.
- What is the Nepal-specific rationale? setting feels data-driven rather than conceptually grounded.

**Theoretical Framework**

- Clear definition of syndemics and alignment with clustering, interaction, and context. Great that this is a novel extension beyond HIV/AIDS.
- Confusing about whether diarrhea is part of the syndemic or a downstream outcome;
- “disadvantaged households” not fully explained;
- Nepal’s gender and caste dynamics could also use more explanation.

**Research Setting & Data Source**

- Good description of Nepal’s political/social background and DHS design.
- Political history feels a bit disconnected from research focus; epidemiological justification perhaps would make more sense for Nepal (IPV, depression, nutrition, diarrhea rates);
- DHS justification makes it sound more like convenience sampling rather than conceptual fit.

**Measures**

- Validated instruments; thoughtful adaptation for vegetarianism and caste; DAG-informed covariates, very detailed and comprehensive.
- The section reads more like a methods appendix. Long technical details (e.g., “Stata’s singleunit(certainty)” option, Taylor series linearization, svyset command) could overwhelm readers. Perhaps move some of the coding/software detail to a supplementary appendix. In the main text, keep it at the level of survey-weighted logistic regression with adjustments for clustering.
- inconsistent treatment of depression (binary vs. continuous);
- single-item diarrhea outcome could present sampling limitations;
- collapsing wealth quintiles needs justification.

**Analytical Strategy**

- Aligned with syndemic theory; tests both additive and multiplicative interaction; sensitivity analyses strengthen robustness. Overall, super strong section.
- Equations/legends could be moved to supplement
- Why are the authors treating depression as continuous?

**Results**

- Rigorous, with stratification, predicted probabilities, and sensitivity analyses, highly detailed.
- Central finding (syndemic effects appear only in disadvantaged households) a bit obscured. The most important finding seems to be: *In disadvantaged households, the syndemic interaction significantly increases child diarrhea risk, but not in advantaged households.* Right now, that message is buried among technical details
- Probability estimates sound quite large given small sample size and wide CIs. The rise from 7.5% to 53.5% predicted probability (lines 486–494) sounds dramatic but could raise concerns about plausibility. The small sample size for triply exposed mothers (noted in earlier results) means CIs are wide — but here you highlight point estimates.

**Discussion & Conclusion**

- Highly comprehensive, policy-relevant, theoretically grounded; offers proportionate universalist strategies and integrates syndemic theory into maternal-child health.
- A bit too long and diffuse; central contribution (context-dependent syndemic effect) should be foregrounded.
- Intervention pathways scattered rather than prioritized. The recommendations are thoughtful but a bit scattered (community health workers, gender-transformative programs, wealth interventions, program integration). Suggestion: Consolidate into 2–3 clear, prioritized pathways: (1) prevention of IPV and treatment of depression, (2) nutrition support, (3) structural inequities (poverty, caste).
- Limitations section overly technical
