# Supplementary material for: Parenting under the triple burden of violence, depression, and poor diet quality: An intergenerational mother–child syndemic in Nepal
Source: PLOS Glob Public Health. 2026 Feb 18;6(2):e0005977. doi: 10.1371/journal.pgph.0005977 (PMC12915984; doi:10.1371/journal.pgph.0005977)
Supplement: Attachment — Submitted filename: Peer_Review_Response_Table.docx [file pgph.0005977.s006.docx]

# Peer Review Response Table

|  | **Comment** | **Author's Reply** | **Evidence of Change in Manuscript** |  |
| --- | --- | --- | --- | --- |
|  | **Editor** | | | |
| 1 | The reviewers and I found the manuscript to be well-written, and to offer a novel application of the syndemics framework to explore complex relationships between child and maternal health in Nepal. The methodology is meticulously described, which is also helpful. Please note that reviewers uploaded their detailed feedback as separate attachments, and please make sure to address those comments in the revisions. | This invitation to revise our work is well received and we are impressed with the quality of feedback we received.  We provided responses to each comment and evidence of relevant manuscript changes. Please let us know if any further information is required. | Please review our response table. |  |
| 2 | Both the reviewers and I found the introduction and the model presented to be a little difficult to follow. Many different topics and variables are presented, with varying degrees of explanation about how they relate to the model(s) to be tested. Streamlining this to focus more clearly on what is presented in Figure 1 and what will be tested would be helpful. | We agree and have revised the introduction accordingly to streamline the topics/variables closer to Figure 1 (our theoretical model). | Introduction and Theoretical framework sections. |  |
| 3 | The methods could also be streamlined, with technical details moved to an appendix, which would make it easier for the reader to understand the overall approach. Reviewers also had several questions about variables that need further explanation.  The results and discussion would also benefit from clearer organization and streamlining as recommended by both reviewers. | Indeed, the methods were overly technical. We have revised this by placing the modelling approach (Equations 1-2) in the appendix. We also responded to the reviewers’ comments about variables and related explanations  Extending streamlining and organization revisions, we have amended the discussion as well. | Supplementary Appendix, Methods, and Discussion sections. |  |
| 4 | Provide separate figure files in .tif or .eps format per journal guidelines. | We added separate figure files. | n/a |  |
| 5 | Clarify funding information in the online submission form and confirm the order of contributions. Indicate funders’ roles in the study. | We clarified the funding information. |  |  |
| 6 | Please send a completed 'Competing Interests' statement, including any COIs declared by your co-authors. If you have no competing interests to declare, please state "The authors have declared that no competing interests exist". Otherwise please declare all competing interests beginning with the statement "I have read the journal's policy and the authors of this manuscript have the following competing interests:" | We completed the ‘Competing interests’ statement | n/a |  |
| 7 | If the reviewer comments include a recommendation to cite specific previously published works, please review and evaluate these publications to determine whether they are relevant and should be cited. There is no requirement to cite these works unless the editor has indicated otherwise. | We have noted this guidance. | n/a |  |
|  | **Reviewer 1** | | | |
| 8 | This study focuses on identifying the relationship between concurrent exposure of intimate partner violence, depression, and inadequate iron intake among mothers on diarrheal disease among children. The study used a national representative sample of women of reproductive age in Nepal. The findings suggest risk of diarrhea is higher in households with concurrent exposure, and that this is further exacerbated by living in a disadvantaged household. The manuscript is well written and very comprehensive with all major topics addressed effectively. The methods present clear description of source of data, procedures used, and approach, providing solid understanding. | Thank you for a careful and detailed assessment of our work. We are grateful for your guidance and hope to have responded with sufficient detail. | No change |  |
| 9 | However, to build a case for examining this association specifically, the relationship of each variable among mothers with the diarrheal disease in children could be more thoroughly explained. What factors specifically influence the connection between depressive symptoms and diarrhea for example? This is not clear enough in Figure 1. | We agree with the importance of building a clearer case for examining the interactions involving child diarrheal disease, maternal intimate partner violence, depression, and nutrition. We have re-organized and streamlined the introduction to add more clarity, in the form of a narrative, for Figure 1.  We opted not to make Figure 1 more complex by adding mediators between the maternal syndemic exposure and the outcome since this analysis does not test mediation pathways and statistical interaction is a separate concept. | Introduction and Theoretical Framework Sections. |  |
| 10 | From the text, it seems like the authors are suggesting it’s parenting practices that connect these two, but I think more evidence would be needed to justify this over some confounding factor like water quality or poverty. | Indeed, parenting is one potential pathway that we describe in the Introduction (though it has not been extensively and directly studied from a syndemic perspective). We added more literature on parenting withing the context of adversities to support our claims. Thank you for raising this issue.  In addition, water quality and water and sanitation infrastructure are indeed confounding variables, which we have held constant our analysis (albeit with the limitation that they are measured imperfectly). We draw attention to these household/environmental factors but emphasize that parenting in an environment where violence, depression, and nutritional insufficiencies affect mothers poses additional challenges to addressing diarrheal disease in children and vulnerability to disease, above and beyond, water quality and water and sanitation infrastructure. However, we understand that residual confounding is still present given we are using observational data. Accordingly, in the Limitations section, we mention our analyses are not causal and should be interpreted as associations. | Introduction section |  |
| 11 | Furthermore, what theoretical mechanisms suggest there is a syndemic effect of these three, related, issues? The use of the theoretical framework is a strength, but the lack of clarity, particularly in the introduction and figure, to justify a hypothesized relationship is a major limitation that limits our enthusiasm considerably. | Indeed, the lack of mechanistic clarity was a limitation. In addressing comment # 9, we draw greater attention and clarity to the theoretical mechanism linking violence, depression, and nutrition insufficiencies among mothers. Specifically, we make the case that healthy parenting in situations of stress is impaired, with downstream effects on child health outcomes such as diarrheal disease. |  |  |
| 12 | Methodology- The binary categorization of iron intake does not capture adequacy. Consider rewording to avoid misinterpretation since adequacy may have different implications than presence/absence of intake alone. In one table, you use no dietary iron intake. I like this better than claims around adequacy, which would be difficult to interpret based on the measure you used. | We agree that the iron consumption measurement approach in the DHS is limited. Our binary operationalization only captures dietary iron intake in the past 24 hours (relative to the time of the survey). For this reason, we cannot conclude ‘no’ dietary intake as the respondent could have consumed iron sources prior to the recall period of 24 hours.  As a compromise, we opted for an amended wording for the iron consumption that we feel addresses the reviewer’s concerns: i) women who reported no dietary iron intake in the past day and ii) absent dietary iron intake (for brevity). | Iron-Rich Food Intake (Previous Day) subsection of the Measures section in the Methods. |  |
| 13 | Line 218 - Maternal syndemic exposures. Can you provide rationale for the temporality of the explored relationship? This is especially important since some of the results imply directionality, yet iron intake is measured on a shorter time horizon than diarrhea symptoms. | The reviewer raises an important point about temporality. As with other secondary analyses conducted using the Demographic and Health Surveys (DHS), the correct temporal order between variables is not guaranteed and often not correct.  We attempted to position exposure and outcome variables logically based on recall periods. Yet, temporality remains a key limitation that we further explain in the discussion. The key assumption we are making regarding the iron consumption recall period is that past 24 hour intake can approximate habitual diet patterns. Given this limitation, we also softened our language concerning directionality. | Maternal Syndemic Exposures subsection of the Measures section in the Methods. |  |
| 14 | Results- Consider streamlining of results section. Since the central theme of the article is triple exposure, creating groups (IPV+Dep+Fe or something like this) and labelling can improve readability. The use of triply/doubly/singly exposed was hard to follow. | This is a fantastic suggestion, which we have incorporated. Thank you! | Throughout the Results and Discussion sections. |  |
| 15 | Discussion- Consider linking current findings to the existent literature to further strengthen discussion. Right now, there is a somewhat limited use of citations and not sufficient connection back to the actual findings of the study.  Much of the section about potential intervention pathways feels premature based on your findings. This may be more noticeable because the mechanism connecting the exposures with the outcome doesn’t feel clear. | We welcome this opportunity to strengthen the discussion.  Accordingly, we: 1) improved connection back to the findings by linking our interpretation to the results (this is in the first 2 paragraphs of the discussion), 2) linking to other scholarly literature, while recognizing the limited empirical studies in LMICs on maternal-child syndemics.  With these revisions, the discussion section now better support the parenting pathways that feature in the discussion. | Paragraphs 1-3 of the Discussion |  |
| 16 | Line 97-98: *These findings indicate a mechanism…via mediators such as diarrheal*. Please add citation/s to support this point. | While editing the introduction, we removed the sentence that appeared on lines 97-98. | n/a |  |
| 17 | Line 105-108: *IPV can lead to…leave children more vulnerable to diarrheal disease.* Please add citation/s to support this point. | While editing the introduction, we removed the sentence that appeared on lines 165-108. | n/a |  |
| 18 | Line 122-123: *Inadequate dietary intake…healthy parenting practices.* Please add citation/s to support this point. | While editing the introduction, we removed the sentence that appeared on lines 122-123 | n/a |  |
| 19 | Line 161-163: *In many LMIC’s…high income counterparts.* Please add citation/s to support this point. | While editing the introduction, we removed the sentence that appeared on lines 161-163. | n/a |  |
| 20 | Line 281: A comma seems to be missing between food insecurity and urban/rural residence | Thank you for bringing this to our attention. We added a comma. | Line 343 |  |
| 21 | Line 397: It is written as “470 of 10,000” instead of 100,000. This appears to be a typographical error and should be corrected for consistency. | Thank you for bringing this to our attention. We corrected the typographical error. | Line 462 |  |
| 22 | Page 23: *Counterfactually, for mothers… (****P=0.00****)…nonsignificant (29.8%, P=0.195).* Could you please correct the p-value and simplify the language for better understanding of this result. We found this to be confusing | We amended the presentation of the p value and simplified the language in question. | Page 26 |  |
| *23* | *Page 29:* GBV is not spelled out. | This has been fixed. | Page 33 |  |
| 24 | Limitations – You mention caste membership and sanitation as covariates in the methods and then discuss it again in the limitations. Please add an explanation of why controlling for these in the analysis was not sufficient. | Additional details concerning caste have been added. | Limitations section of the Discussion |  |
|  | **Reviewer 2** | | | |
| 25 | This manuscript addresses an important and novel question by applying syndemic theory to maternal and child health in Nepal. The study uses nationally representative DHS data and rigorous interaction modeling to explore how IPV, maternal depression, and inadequate dietary iron intake cluster and interact to affect child diarrhea outcomes. The theoretical contribution is strong, and the policy relevance is clear. | Thank you for this careful and detailed read and assessment of our work. | n/a |  |
| 26 | However, the manuscript could do with significant tightening (moving material to an appendix), stronger contextual justification, and clearer alignment between theory, exposures, and outcomes. | The reviewer addresses many important and well received points, which we have addressed below. | Changes made in multiple parts of the manuscript, as explained below. |  |
|  | **Abstract** |  |  |  |
| 27 | Novel application of syndemic theory; well-structured research questions; strong methodological rigor. | Thank you | n/a |  |
| 28 | Diarrhea is introduced abruptly and not adequately justified as the outcome; conceptual drift between “child health disparities” broadly and “diarrhea” specifically.  Dramatic probability increases need context (CIs, effect sizes). A bit confusing – would benefit from more clarity. | We better framed diarrheal symptoms as one possible child health outcome wherein the population prevalence is inequitably distributed.  In the revised abstract, we added the estimated coefficient for the interaction terms on multiplicative and additive scales. We opted to present interaction term coefficient results instead of the model predicted probabilities because estimated coefficients are more common in the literature and the predicted probabilities have low precision (as we explain later on in the results section). | Abstract |  |
|  | **Introduction & Literature Review** |  |  |  |
| 29 | Broad, well-referenced coverage of IPV, depression, nutrition, and gender inequities. | Thank you | n/a |  |
|  | The first two paragraphs (lines 68–87) pack in too much — global child mortality, diarrheal disease burden, services, parenting roles, and gender inequity — without clearly signaling the specific *research problem*. It feels like you are setting up multiple possible papers: one on diarrhea epidemiology, another on maternal mental health, another on gendered nutrition inequities. It could help to condense the background to 2–3 crisp points: (1) diarrhea is a major child mortality driver in LMICs, (2) maternal health and caregiving context strongly influence child vulnerability, (3) three maternal adversities (IPV, depression, inadequate nutrition) may jointly increase risk. | The reviewer raises an important point about condensing the background to emphasize the 3 main points. We appreciate these suggestions and have incorporated them. | First paragraph of the Introduction. |  |
| 30 | need more justification for diarrhea vs. other outcomes;  maternal exposures reviewed in parallel instead of as an interconnected system. | The theoretical framework we present in Figure 1 can be adapted to other child health and development outcomes as well as other maternal syndemic exposures. Our decision to analyze diarrheal disease (rather than other child health and development outcomes) was made based on data availability. In this way, our focus on Nepal can be seen as a case study where we apply the theoretical framework outlined in Figure 1.  The maternal exposures of violence, dietary intake, and depression are interconnected through a web of potential mechanisms. We revised the introduction to better explain these mechanisms. Though, many such mechanisms have not been robustly and directly examined. | Theoretical Framework section of the Introduction |  |
| 31 | What is the Nepal-specific rationale? setting feels data-driven rather than conceptually grounded. | The review is correct- our focus on Nepal was purely data driven based on data availability. Since we did not collect any primary data to test the conceptual model, we relied on existing data from the Demographic and Health Surveys. We now explain this approach in the manuscript. | Theoretical Framework section of the Introduction. |  |
|  | **Theoretical Framework** |  |  |  |
| 32 | Clear definition of syndemics and alignment with clustering, interaction, and context. Great that this is a novel extension beyond HIV/AIDS. | Thank you | n/a |  |
|  | Confusing about whether diarrhea is part of the syndemic or a downstream outcome; | Diarrheal disease is the outcome. We labeled this in the improved figure. | Figure 1 |  |
| 33 | “disadvantaged households” not fully explained; | We welcome this opportunity to better explain disadvantage. This information has been added to the revised manuscript. | Theoretical framework section as well as the Methods section |  |
| 34 | Nepal’s gender and caste dynamics could also use more explanation. | We agree with the reviewer and have added this information to the country research setting. | Research setting subheading of the Methods. |  |
|  | **Research Setting & Data Source** |  |  |  |
| 35 | Good description of Nepal’s political/social background and DHS design. | Thank you | n/a |  |
| 36 | Political history feels a bit disconnected from research focus; epidemiological justification perhaps would make more sense for Nepal (IPV, depression, nutrition, diarrhea rates);  DHS justification makes it sound more like convenience sampling rather than conceptual fit. | Regarding the county context section, we feel it is important to mention Nepal’s political history to add a rich contextual backdrop to the interpretation and potential policy / program interventions.  Indeed, our choice to test the theoretical model presented in Figure 1 was grounded in data availability. In responding to reviewer comment #31, we have also addressed this comment. However, kindly note that the DHS does not use convenience sampling; it is a nationally representative probability sample, which supports both the internal and external validity of our analysis. | Research setting subsection of the Introduction  Theoretical Framework section of the Introduction. |  |
|  | **Measures** |  |  |  |
| 37 | Validated instruments; thoughtful adaptation for vegetarianism and caste; DAG-informed covariates, very detailed and comprehensive. | Thank you | n/a |  |
| 38 | The section reads more like a methods appendix. Long technical details (e.g., “Stata’s singleunit(certainty)” option, Taylor series linearization, svyset command) could overwhelm readers. Perhaps move some of the coding/software detail to a supplementary appendix. In the main text, keep it at the level of survey-weighted logistic regression with adjustments for clustering. | We agree with the reviewer that the methods section was overly technical. We added the details mentioned to the appendix. | Supplementary Appendix B |  |
| 39 | inconsistent treatment of depression (binary vs. continuous); | We used 2 operationalizations for depression because we had different analysis aims. For descriptive analysis we used a categorical operationalization, which allowed us to compute estimates of clustering prevalence. For inferential analysis we used a numeric operationalization for the PHQ depression scale; this maximized power and improved model fit since the depression scale is normally numeric. | Depression symptoms (previous 2 weeks) of the Measures section in the Results. |  |
| 40 | single-item diarrhea outcome could present sampling limitations; | Indeed, the diarrheal measurement approach used in the DHS is limited. We detail these limitations in the discussion. | Limitations section |  |
| 41 | collapsing wealth quintiles needs justification. | We agree with the reviewer and have added a justification. Briefly, our analysis could not support stratification by all 5 wealth levels (poorest, poor, middle, least poor, poorest). With each stratification, we limit the sample size available to run a complex interaction model involving three term interactions. Thus, we collapsed the wealth quintile to isolate the 2 lowest levels of wealth and middle wealth and higher. Though, we recognize this approach is limited in that we cannot detect any dose-response relationships. We note this limitation in the discussion. | Contextual Variable (Household wealth) subsection of the Measures section from the Results. |  |
|  | **Results** |  |  |  |
| 42 | Rigorous, with stratification, predicted probabilities, and sensitivity analyses, highly detailed. | Thank you | n/a |  |
| 43 | Central finding (syndemic effects appear only in disadvantaged households) a bit obscured. The most important finding seems to be: *In disadvantaged households, the syndemic interaction significantly increases child diarrhea risk, but not in advantaged households.* Right now, that message is buried among technical details  Probability estimates sound quite large given small sample size and wide CIs. The rise from 7.5% to 53.5% predicted probability (lines 486–494) sounds dramatic but could raise concerns about plausibility. The small sample size for triply exposed mothers (noted in earlier results) means CIs are wide — but here you highlight point estimates. | We thank the reviewer for suggesting better language to draw attention to our main findings. We have applied this to our revised manuscript.  The reviewer raises an important point about precision, which we agree with. We added CI to the estimated model predicted probabilities; this allows the reader to assess the precision of the findings in addition to the magnitude of the predicted probabilities. On a broader note, we use the predicted probabilities as one tool to describe the interaction patterns. We also have Models 1a, 1b, 2a, and 2b as well as the marginal predicted probability plots. Thus, the predicted probabilities should be interpreted alongside the other analyses that describe the interaction patterns. | Syndemic Interactions and Context subsection of the Results |  |
|  | **Discussion & Conclusion** |  |  |  |
| 44 | Highly comprehensive, policy-relevant, theoretically grounded; offers proportionate universalist strategies and integrates syndemic theory into maternal-child health. | Thank you | n/a |  |
| 45 | A bit too long and diffuse; central contribution (context-dependent syndemic effect) should be foregrounded. | We agree and have paid greater attention to the central contribution of our analysis. | First two paragraphs of the Discussion |  |
| 46 | Intervention pathways scattered rather than prioritized. The recommendations are thoughtful but a bit scattered (community health workers, gender-transformative programs, wealth interventions, program integration). Suggestion: Consolidate into 2–3 clear, prioritized pathways: (1) prevention of IPV and treatment of depression, (2) nutrition support, (3) structural inequities (poverty, caste). | Thank you for this opportunity to improve our discussion. We appreciate the suggestion to consolidate and have applied this accordingly. | Parenting Pathways section of the Discussion |  |
| 47 | Limitations section overly technical | We have simplified the language in the limitations sections to reduce the technical tone. | Limitations section of the Discussion. |  |
