# Supplementary material for: Parenting under the triple burden of violence, depression, and poor diet quality: An intergenerational mother–child syndemic in Nepal
Source: PLOS Glob Public Health. 2026 Feb 18;6(2):e0005977. doi: 10.1371/journal.pgph.0005977 (PMC12915984; doi:10.1371/journal.pgph.0005977)
Supplement: Attachment — Submitted filename: plos_global_review2.docx [file pgph.0005977.s005.docx]

I appreciate the modifications made by the authors to address reviewer comments. I think the improvements significantly strengthened the manuscript. I have no further major comments. Below are a few small grammatical errors that should be corrected:

- Line 87 – maternal is used twice in that sentence
- Line 102- consider rewording “clean water and water and sanitation infrastructure” to improve clarity
- Page 25, first sentence of second paragraph: It seems like a word might be missing after “empirical”
