# Supplementary material for: Parenting under the triple burden of violence, depression, and poor diet quality: An intergenerational mother–child syndemic in Nepal
Source: PLOS Glob Public Health. 2026 Feb 18;6(2):e0005977. doi: 10.1371/journal.pgph.0005977 (PMC12915984; doi:10.1371/journal.pgph.0005977)
Supplement: Attachment — Submitted filename: R2 Response to reviewers.docx [file pgph.0005977.s007.docx]

Manuscript Title: Parenting Under the Triple Burden of Violence, Depression, and Poor Diet Quality: An Intergenerational Mother–Child Syndemic in Nepal

Manuscript ID: PGPH-D-25-01038R1

Revision due date: Feb 12, 2026

| **Comment** | **Author Response** | **Evidence of change to manuscript** |
| --- | --- | --- |
| Editor comments:   - The reviewers and I appreciate the authors' responsiveness to the previous reviews and feel that the manuscript is much clearer. - However, there are several areas where minor errors and inconsistencies need to be clarified (see below and attached for specific notes). | Thank you for considering our manuscript. We are happy to resolve these minor issues and have made the appropriate changes. | n/a |
| Reviewer 1 comments:  I appreciate the modifications made by the authors to address reviewer comments. I think the improvements significantly strengthened the manuscript. I have no further major comments. Below are a few small grammatical errors that should be corrected:   - Line 87 – maternal is used twice in that sentence - Line 102- consider rewording “clean water and water and sanitation infrastructure” to improve clarity - Page 25, first sentence of second paragraph: It seems like a word might be missing after “empirical” | We have corrected the small grammatical errors on lines 87, 102, and Page 25. | 1. Line 87: Mechanisms between such harmful maternal exposures and child diarrheal disease have not been robustly established in empirical literature. 2. Line 102: We hypothesize that these interactions related to stress and parenting are magnified in more disadvantaged households, above and beyond the presence of clean water and adequate sanitation infrastructure. 3. Page 25: Through other scholarship has conceptually positioned syndemics as a useful framework for child health and development inequities such as diarrheal disease [38, 69–71], empirical scholarship is only yet emerging. |
| Reviewer 2 Comments:   This study makes a strong contribution by advancing an intergenerational syndemic framework linking maternal intimate partner violence (IPV), depressive symptoms, and nutritional vulnerability to child diarrheal illness in Nepal. The syndemic framing is clear and theoretically grounded, with explicit objectives addressing clustering, interaction, and contextual forces. The analysis appropriately uses DHS data, accounting for survey design and the domestic violence module, and the interaction testing is carefully implemented using fully saturated models on both additive and multiplicative scales, supported by predicted probabilities. Limitations related to cross-sectional design, measurement, and statistical power are acknowledged transparently. | Thank you for your detailed assessment of our manuscript. |  |
| Several clarifications would strengthen alignment between claims and measures. Most notably, the nutrition exposure reflects reported consumption of iron-rich foods in the prior 24 hours instead of rather than malnutrition or iron deficiency, and should be framed as a short-term dietary quality indicator. | We agree that the nutrition exposure is a measure of short-term diet quality, rather than malnutrition. To ensure greater alignment between the claims and measures, we replaced the term ‘malnutrition’ with ‘poor diet quality’ and explained that the minimum dietary diversity tool is a measure of short term diet quality. | See changes throughout the manuscript, as well as in the title, which now reads: “Parenting Under the Triple Burden of Violence, Depression, and Poor Diet Quality: An Intergenerational Mother–Child Syndemic in Nepal.”  Methods section:  ***Iron-Rich Food Intake (Previous Day):*** Women’s dietary intake, a measure of short-term diet quality, was assessed using The Minimum Dietary Diversity for Women (MDD-W) tool. |
| In addition, the abstract appears to reverse additive and multiplicative interaction estimates (with the odds ratio and linear probability coefficient mislabeled), which should be corrected for consistency with Table 2. | The reviewer is correct in that we made an error in reporting the estimates for the additive versus multiplicative interactions regarding the abstract. | Abstract:  In disadvantaged households (two lowest household wealth quintiles), children’s odds of diarrhea were higher when mothers experienced syndemic vulnerability (IPV, depression, and absent iron intake) on multiplicative (aOR=1.321, 95%CI:1.000, 1.745) and additive scales (B=0.027, 95%CI:-0.002, 0.055). |
| Minor typographical errors in the tables should also be addressed. { Your key interaction estimates are confusingly reported (OR vs B swapped in text). In the abstract you report additive B=1.321 and multiplicative B=0.027, but in Table 2: • Multiplicative: OR for 3-way term is 1.321 • Additive (LPM): B for 3-way term is 0.027  So the abstract has the scales/labels reversed. | We have addressed the inconsistent reporting of B and OR such that the estimates from the multiplicative interaction model (logistic regression) are odds ratios and estimates from the additive interaction model (OLS regression) are reported as B. | Abstract:  In disadvantaged households (two lowest household wealth quintiles), children’s odds of diarrhea were higher when mothers experienced syndemic vulnerability (IPV, depression, and absent iron intake) on multiplicative (aOR=1.321, 95%CI:1.000, 1.745) and additive scales (B=0.027, 95%CI:-0.002, 0.055). |
| Additional typos “10.40 (9.16, 1180)” → likely “11.80” “2.01 (1.52. 2.51)” → comma typo “1.75 (0.0082, 3.69)” → likely “0.82” not “0.0082” } | Thank you for the attention to detail. We have corrected each typo in Table 1. | See the revised Table 1. |
| Interpretation of the findings should remain cautious given discordant recall periods across exposures and outcomes, the rarity of concurrent exposure to all three maternal adversities, and reliance on a single maternal report of child diarrhea. These factors likely contribute to imprecision in three-way interaction estimates and wide confidence intervals for some predicted probabilities, which are best viewed as illustrative of interaction patterns rather than precise risk estimates. | We completely agree with the reviewer and have edited the Discussion and Limitations accordingly. | See revised Discussion section, which softens the causal language/assumptions regarding the policy implications stemming from our results.  Limitations:  Finally, due to several methodological constraints (non temporally aligned recall periods between the exposures and the outcome, the low prevalence of simultaneous exposure to all three maternal adversities, and dependence on a single maternal report of child diarrhea) causal interpretation of our results warrants caution. Together, these constraints likely reduced precision in the three-way interaction analyses, resulting in wide confidence intervals for certain predicted probabilities. Accordingly, the present estimates should be interpreted as indicative of potential interaction patterns rather than as precise measures of risk. |
| Despite these limitations, the consistency of results across modeling approaches and sensitivity analyses suggests that combined maternal vulnerabilities could meaningfully elevate child diarrhea risk in socioeconomically disadvantaged households. | We agree with this assessment. | n/a |
| Overall, this is a very strong study and it highlights the value of a syndemic framework for identifying contexts in which multiple maternal adversities interact to amplify child health inequities, while underscoring the need for longitudinal and mixed-methods research to clarify mechanisms and causal pathways. | Thank you for your positive assessment. | n/a |
